# What Is the Voluntary Principle ?

**Published:** 1924-07

**Authors:** Ahrdub Luaich


### CORRESPONDENCE.

### What is the Voluntary Principle?

*To the Editor of* The Hospital and Health Review.

Sir,—At the risk of being thought by some to be asking a merely foolish question, or by others to be indulging in a little captious exercise, may I ask for enlightenment? What exactly is the Voluntary Principle, or Voluntaryism, as applied to the work of our hospitals? I know that it is that exquisite something which pervades and permeates the whole of our hospital work, and that it is more or less taken for granted, like sunlight and air. But is it capable of precise definition?

Does the Voluntary Principle, for example, derive its inspiration from the fact that the funds are contributed voluntarily, which after all means that the care of the sick and suffering is a burden falling in a most unequal way on the community at large? And do not these funds only flow at all in some instances as the result of a good deal of coaxing or coercion? Or does the Voluntary Principle merely imply control by persons giving voluntary service? Is it clear that a service of hospitals controlled and co-ordinated by the State would have unfortunate reactions on the spirit in which the patients are attended?

Or, finally, does Voluntaryism owe its inspiration to the fact that medical services are usually given gratuitously? Are there no attendant advantages to the doctor? So far as surgeons and other specialists are concerned, is their income not assured to them by a heavier range of fees in their private practice than would be in operation if there were no gratuitous hospital work? In other words, does not the burden again fall unequally on the community?

Ahrdub Luaich.

